# Distinguishing Obstructive Versus Central Apneas in Infrared Video of Sleep Using Deep Learning: Validation Study

**DOI:** 10.2196/17252

**Published:** 2020-05-22

**Authors:** Sina Akbarian, Nasim Montazeri Ghahjaverestan, Azadeh Yadollahi, Babak Taati

**Affiliations:** 1 Kite Research Institute, Toronto Rehabilitation Institute University Health Network Toronto, ON Canada; 2 Institute of Biomaterials & Biomedical Engineering University of Toronto Toronto, ON Canada; 3 Vector Institute Toronto, ON Canada; 4 Department of Computer Science University of Toronto Toronto, ON Canada

**Keywords:** noncontact monitoring, sleep apnea, motion analysis, computer vision, obstructive apnea, central apnea, machine learning, deep learning

## Abstract

**Background:**

Sleep apnea is a respiratory disorder characterized by an intermittent reduction (hypopnea) or cessation (apnea) of breathing during sleep. Depending on the presence of a breathing effort, sleep apnea is divided into obstructive sleep apnea (OSA) and central sleep apnea (CSA) based on the different pathologies involved. If the majority of apneas in a person are obstructive, they will be diagnosed as OSA or otherwise as CSA. In addition, as it is challenging and highly controversial to divide hypopneas into central or obstructive, the decision about sleep apnea type (OSA vs CSA) is made based on apneas only. Choosing the appropriate treatment relies on distinguishing between obstructive apnea (OA) and central apnea (CA).

**Objective:**

The objective of this study was to develop a noncontact method to distinguish between OAs and CAs.

**Methods:**

Five different computer vision-based algorithms were used to process infrared (IR) video data to track and analyze body movements to differentiate different types of apnea (OA vs CA). In the first two methods, supervised classifiers were trained to process optical flow information. In the remaining three methods, a convolutional neural network (CNN) was designed to extract distinctive features from optical flow and to distinguish OA from CA.

**Results:**

Overnight sleeping data of 42 participants (mean age 53, SD 15 years; mean BMI 30, SD 7 kg/m^2^; 27 men and 15 women; mean number of OA 16, SD 30; mean number of CA 3, SD 7; mean apnea-hypopnea index 27, SD 31 events/hour; mean sleep duration 5 hours, SD 1 hour) were collected for this study. The test and train data were recorded in two separate laboratory rooms. The best-performing model (3D-CNN) obtained 95% accuracy and an *F*_1_ score of 89% in differentiating OA vs CA.

**Conclusions:**

In this study, the first vision-based method was developed that differentiates apnea types (OA vs CA). The developed algorithm tracks and analyses chest and abdominal movements captured via an IR video camera. Unlike previously developed approaches, this method does not require any attachment to a user that could potentially alter the sleeping condition.

## Introduction

### Background

Sleep apnea is a chronic respiratory disorder, caused by intermittent reduction (hypopnea) or cessation (apnea) of respiratory airflow during sleep. About 10% of the population have this disorder [1], and it increases the risk of heart disease by 3-fold, stroke by 4-fold, and car accidents by 7-fold [2-5]. The severity of sleep apnea is commonly measured via the apnea-hypopnea index (AHI), which shows the number of apneas and hypopneas per hour of sleep.

Depending on the presence of breathing effort, sleep apneas can be divided into obstructive sleep apnea (OSA) or central sleep apnea (CSA) by measuring the thoracoabdominal movement and its contributions to the total respiratory volume [6]. The majority of sleep apneas are obstructive [7], which is caused by the full collapse of the pharyngeal airway that blocks the flow of air into the lungs [8]. The rest of sleep apneas are central, which happen due to a reduction in the respiratory drive from the central nervous system [9].

**Figure 1:**
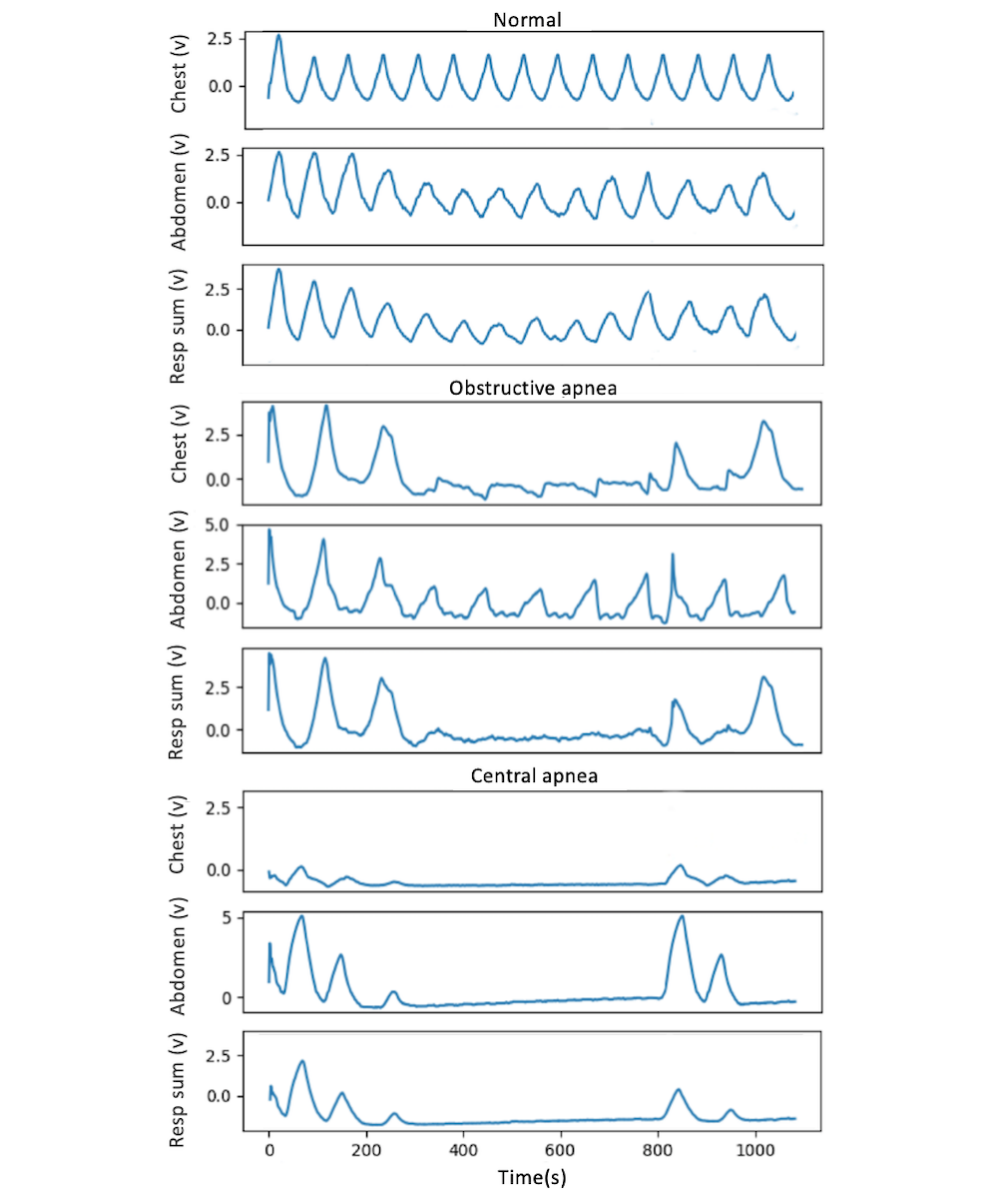
Difference between the movements of the chest and abdomen and the sum of the two movements during obstructive apnea (OA), central apnea (CA), and normal breathing. During normal breathing, chest and abdomen movements are in phase. During OA, breathing effort and airway blockage result in the out-of-phase movement of the chest and abdomen, and the sum of the two movement signals (respiratory sum) is close to zero. During CA, there is no movement in the chest or the abdomen. CA: central apnea; OA: obstructive apnea; Resp sum: respiratory sum.

During normal breathing, the chest and abdomen move in phase due to interaction between the diaphragm and parasternal intercostals during inhalation [10]. During obstructive apnea (OA), airway obstruction leads to an out-of-phase motion of the rib cage and the abdomen, causing a reduction in the sum of chest and abdomen’s movement [6]. On the other hand, during central apnea (CA), there is no movement in the rib cage or the abdomen due to lack of brain signal for muscle contraction. Figure 1 compares the movements in the chest and the abdomen and the sum of them during OA, CA, and normal breathing. If the majority of events in a person are obstructive, they will be diagnosed as OSA and otherwise as CSA. In addition, as it is challenging and highly controversial to divide hypopneas into central or obstructive, the decision about sleep apnea type (OSA vs CSA) is made based on apneas only.

The treatment of sleep apnea decreases patients’ health-related costs by 25% [11]. A highly effective treatment for OSA is continuous positive airway pressure (CPAP) therapy that involves applying positive pressure of air to keep the airway open during sleep. However, CPAP therapy leads to increase mortality in patients with CSA [12]. Therefore, a crucial step for proper treatment of sleep apnea is to differentiate OSA from CSA [13].

### Problem Statement

The gold standard for differentiating OA from CA is the evaluation of swings in esophageal pressure measured via an esophageal catheter [14]. Measurement of esophageal pressure is invasive, uncomfortable, and could disrupt sleep. Therefore, esophageal pressure measurements are only used for physiological research purposes [15,16]. The current clinical approach to distinguish OA from CA based on the different patterns of movement in the chest and abdomen is respiratory inductance plethysmography (RIP). It measures thoracoabdominal movements from two transducer bands over the chest and the abdomen. RIP is a part of polysomnography (PSG), which consists of connecting more than 20 sensors to patients. PSG is inconvenient during sleep, expensive (>US $400 in Canada), has long waiting time (4-36 months in Canada [17]), and requires a trained technician to manually analyze the recorded signals, including RIP.

A promising approach to distinguish different types of sleep apnea is using computer vision systems. The goal of computer vision is to extract information from images or videos. In this study, a noncontact approach was proposed to identify the types of apneas (OA vs CA) using computer vision techniques. In this approach, the upper body respiratory movements were captured in infrared (IR, *night vision*) video frames and were processed via computer vision algorithms.

Computer vision systems have been previously developed for various applications in sleep monitoring, for example, to monitor the breathing rate and pulse [18-20], to estimate the AHI [21], to screen sleep quality and irregularities [22,23], and to monitor sleep positions [24]. But the use of computer vision to distinguish different types of apnea (OA vs CA) was unexplored and was the main novel contribution of this study.

## Methods

### Data Collection

Adults aged 18 years or above who were referred to the sleep laboratory of the Toronto Rehabilitations Institute-University Health Network for sleep screening were recruited for this study. The University Health Network Research Ethics Board approved this study (Research Ethics Board approval number 13-7210-DE). Participants signed a written consent form before taking part in the study.

Full overnight PSG was recorded for clinical diagnosis of sleep apnea in two separate rooms. Additionally, an IR camera (Firefly MV, 0.3 MP, FMVU- 03MTM, FLIR Systems) was mounted about 1.4 m above the bed. The rooms were illuminated by IR light (Raytec RM25-F-50). Simultaneous and synchronized with PSG, IR videos were recorded from an overhead view with the aforementioned camera at a resolution of 640×480 at 30 frames per second. The 680×480 resolution captured the upper body part (the head, chest, and abdomen) with sufficient detail.

OA and CA were annotated by 3 trained individuals based on the RIP signal of the chest, the abdomen, the sum of the movement of the chest and the abdomen, nasal pressure, and oxygen saturation, following the American Academy of Sleep Medicine guideline [25,26]. The first 2 experts annotated all data with the agreement of 80%. The third expert annotated the discrepancies.

### Data Analysis

Recorded IR videos were down-sampled from 30 Hz to 2 Hz to decrease the computational cost. The breathing rate during sleep is between 12 and 25 breaths per minute, that is, below 0.5 Hz. Therefore, the reduced sampling frequency of 2 Hz still satisfied the Nyquist rate for the respiratory signal by a wide margin. As each apnea lasts a minimum of 10 seconds, the sampling rate of 2 frames per second translates to a minimum of 20 image frames per event.

Frame-to-frame movements were tracked using dense optical flow. FlowNet 2.0 [27], a convolutional neural network (CNN) model, was used for this purpose. Optical flow generates a 2-channel image comprising the *x* (side to side) and the *y* (up and down) movement vector of each pixel from one video frame to the next. A 3D-CNN model was trained to analyze the sequence of optical flow images during each apnea to distinguish between OAs and CAs. This 3D-CNN model processed the optical flow of the entire image without explicit knowledge of where the person was at every frame of the video. For comparison, another CNN architecture was used to analyze the optical flow sequences in only the chest and abdomen regions. This model was trained to distinguish OAs from CAs via a late fusion of two 3D-CNN branches processing chest and abdominal movements. Performance was evaluated when either manually marked or automatically detected chest and abdomen regions were used. The performance of these CNN architectures was compared with three baseline models, including autocorrelation, movement histograms, and 2-dimensional fast Fourier transform (2DFFT)-CNN, which were chosen as commonly used approaches for motion analysis. These models were not applied previously for distinguishing OAs from CAs. The first two baseline models (autocorrelation and movement histograms) operate on the time-series data of optical flow movements, whereas the third baseline model (2DFFT-CNN) first transforms the signal into the frequency domain and then applies a CNN model to the resulting 2DFFT images.

#### 3D Convolutional Neural Network

Sleep apneas last a minimum of 10 seconds [28]. A 3D-CNN model was trained on a sliding window of 10 seconds (20 frames at 2 Hz), starting from 5 seconds before the start of the apnea until 5 seconds after its ending. The size of the input tensor to the 3D-CNN model is 640×480×2×20 (image size 640×480 pixels; number of channels: 2; number of frames: 20). The two channels are outputs of the optical flow image, indicating the changes in the *x* (side to side) and *y* (up and down) directions. Details of the 3D-CNN model are shown in Table 1. RMSProp was used to optimize a class-weighted cross-entropy loss. The Matthews correlation coefficient was used for early stopping. An initial value of 0.001 for the learning rate and 1000 epoch with the batch size of 32 were used. The total number of parameters in this network was 95,649 of which 95,393 were trainable, and 256 were nontrainable.

OAs are more frequent than CAs; therefore, to achieve a more balanced training set, different stride was using for the sliding window in OAs and CAs (stride of 1 second for CA and stride of 5 seconds for OA). In test time, a stride of 1 second was used, and voting (overall strides within an event) determined the estimated label (OA vs CA) for that event.

**Table 1:** Architecture of a 3D convolutional neural network used to distinguish obstructive apnea from central apnea.

Layer	Number of filters, n	Size/stride	Activation function	Output size
Input	N/A^a^	N/A	N/A	480×640×20×2
Average pool	N/A	25×25×1/20×20×1	N/A	23×31×20×2
Convolutional	8	2×2×1/1×1×1	Linear	22×30×20×8
Dropout	N/A	N/A	N/A	22×30×20×8
Convolutional	16	3×3×5/1×1×1	N/A	20×28×16×16
Max pool	N/A	8×8× /2×2×1	N/A	7×11×16×16
Batch normalization	N/A	N/A	Leaky Relu^b^	7×11×16×16
Convolutional	64	2×2×2/1×1×1	N/A	6×10×15×64
Batch normalization	N/A	N/A	Leaky Relu	6×10×15×64
Convolutional	32	4×4×1/1×1×1	N/A	3×7×15×32
Batch Normalization	N/A	N/A	Relu	3×7×15×32
Dropout	N/A	N/A	N/A	3×7×15×32
Convolutional	16	2×2× /1×1×1	N/A	2×6×15×16
Batch normalization	N/A	N/A	Relu	2×6×15×16
Flatten	N/A	N/A	N/A	2880
Fully connected	16	2880×16	N/A	16
Fully connected	4	16×4	N/A	4
Output layer	N/A	4×1	Sigmoid	1

^a^N/A: not applicable.

^b^ReLu: rectified linear unit.

#### 3D Convolutional Neural Network (Chest and Abdomen)

To investigate if knowledge about the location of the chest and abdomen at each image frame improves performance, the frames were manually marked as follows: for each person, at the first video frame of their sleep. After each position shift, a human annotator manually marked the locations of the chest and abdomen via two rectangles on the image. If the participant was covered by a blanket, the annotator used his/her best judgment to mark these locations (based on the current image frame and also by looking at previous and future image frames). Position shifts were automatically detected based on the total amount of movement in the scene, with a low threshold, so even small position shifts were not missed.

As the annotation of the chest and abdomen regions is subjective and time-consuming, we have also developed an algorithm to automatically find the chest and abdomen regions. To develop this model, as the chest and abdomen were often covered by a blanket sheet, it was challenging to train an object-detection CNN to detect them directly. Instead, a CNN model (YOLO v3) [29] was used first to locate the head, and another model was subsequently used to infer the position of the chest and abdomen bounding boxes. Specifically, a random forest regression model was trained based on the estimated head location, BMI, weight, height, head position (supine vs lateral), and body position (supine vs lateral) to estimate the bounding box of the chest and abdomen. The head and body positions were obtained automatically via a CNN-based model that was previously developed and validated [24].

**Figure 2:**
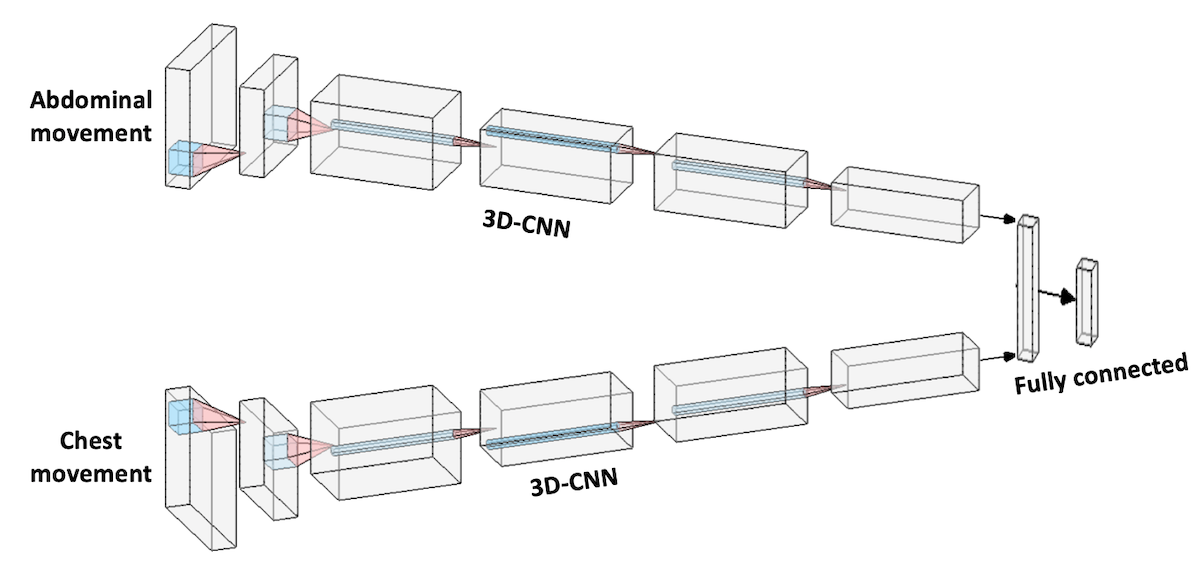
The Convolutional neural network architecture used to extract and combine information from movements of the chest and the abdomen. 3D-CNN: 3D convolutional neural network; CNN: convolutional neural network.

Two 100×100 images were cropped around the estimated center of the chest and abdomen. Dense optical flow was computed in cropped regions of both the chest and abdomen. A 3D-CNN branch processed the sequence of chest movements, and another branch processed the sequence of abdominal movements. Outputs from both branches were concatenated into a fully connected network. The network was trained end to end. The architecture of the entire network is shown in Figure 2. The architecture of the 3D-CNN model in each branch is identical to the one shown in Table 1. The only difference is the first average pooling layer, which has the size and stride of 10 x 10 and 5 x 5, because of the smaller image size.

#### Baseline 1—Autocorrelation

**Figure 3:**
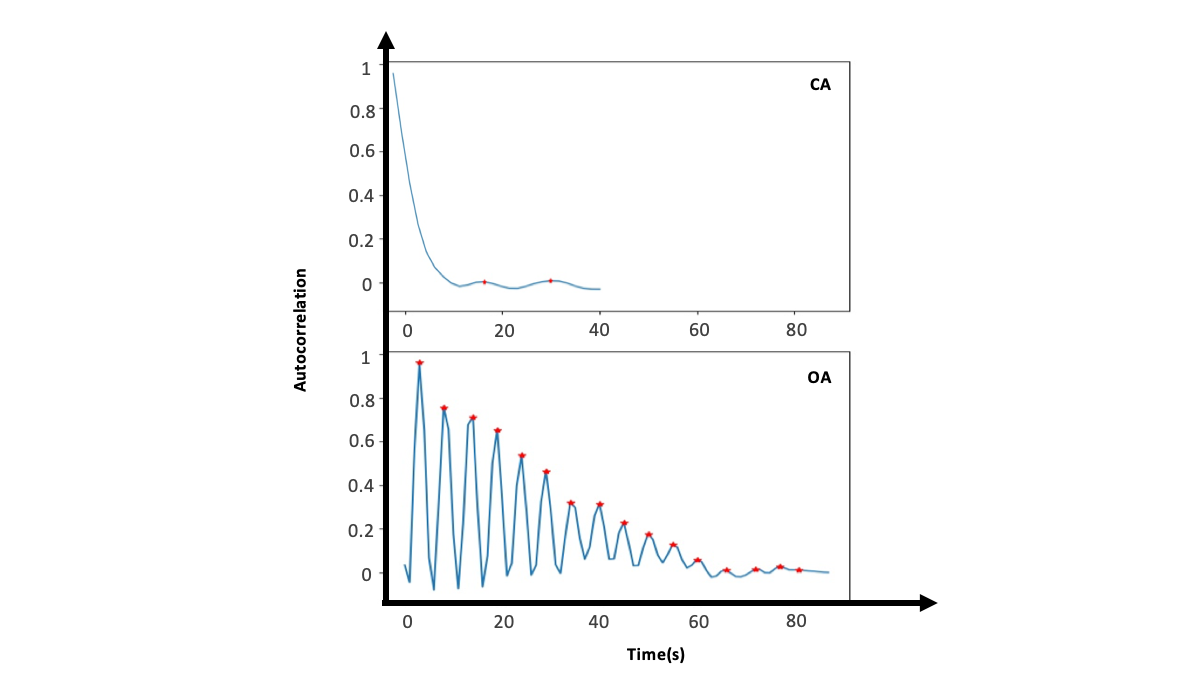
Autocorrelation signal of the movement in an obstructive apnea (OA) and central apnea (CA). OAs are more periodic due to the existence of breathing effort as compared with CAs. Therefore, the OA autocorrelation signal has more peaks, as indicated by red stars. CA: central apnea; OA: obstructive apnea.

This method uses autocorrelation to separate OAs from CAs based on their periodicity. The magnitude of the movement vector (*d*=√(*x*^2^+*y*^2^)) was calculated from the original 2-channel optical flow image (640×480×2) to form a single-channel movement magnitude image (640×480). Pixels with a large movement magnitude (>0.5 pixel per second), likely caused by large position shifts, were capped, that is, set to 0.5. The average of the movement image was then calculated for each event, leading to a 1-dimensional movement signal *m*(*t*). A Butterworth band-pass filter with a lower cutoff frequency of 0.05 Hz and an upper cutoff frequency of 0.5 Hz was applied to the one-dimensional movement signal. Autocorrelation was computed for the filtered signals, and its first 10 peaks (if peaks did not exist, 0 was considered) were used to train three different binary classifiers to distinguish between OA and CA. Classifiers compared were linear support-vector machines, logistic regression, and random forest. Sample autocorrelation signals with their detected peaks are illustrated in Figure 3 for a CA and an OA.


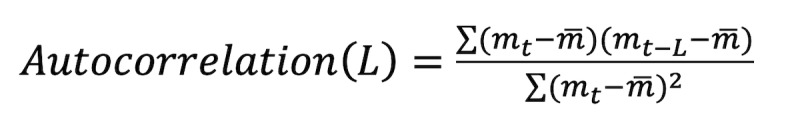
 is autocorrelation, where *m_t_* is an event signal, is an average of signal, and is a shifted event with a lag of *L*. Lag (*L*) was set equal to the event duration. The summations are over all the values of *t*.

#### Baseline 2—Movement Histograms

**Figure 4:**
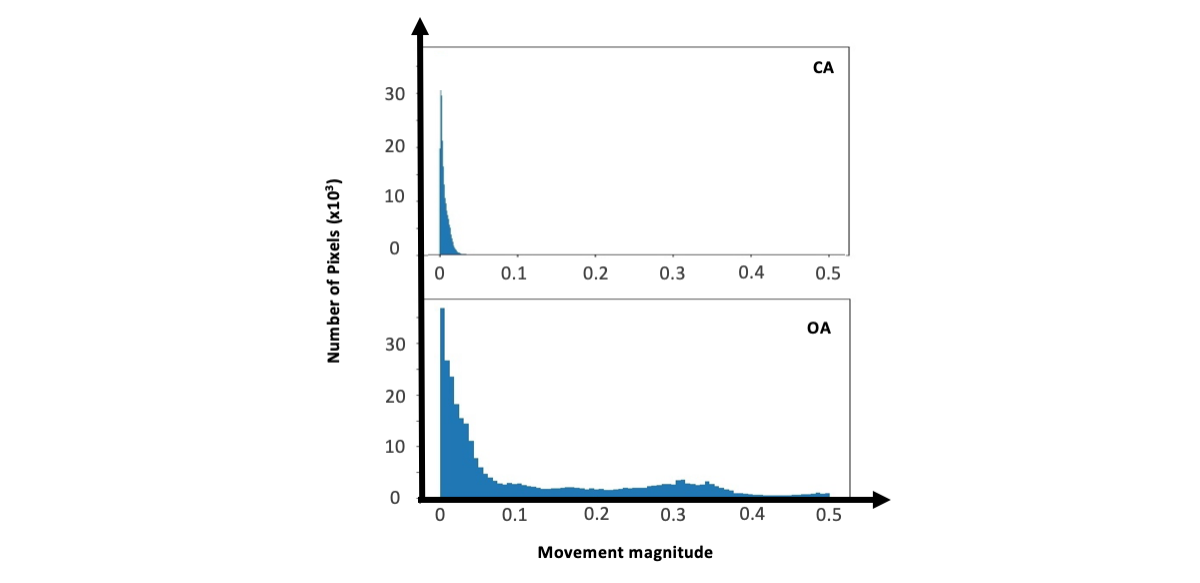
Histogram of movement magnitudes. Obstructive apneas have more range of motion as compared with central apneas because of the breathing effort. CA: central apnea; OA: obstructive apnea.

This method separates OAs from CAs based on their range of motion. Histogram of the movement magnitude was constructed for movements in the range of 0 to 0.5 pixels/second with a constant number of 1000 bins. Sample histogram signals for OA and CA are shown in Figure 4. The average of each bins (features) of histogram across an event was computed. Principal component analysis (PCA) was subsequently applied to reduce the number of bins. A random forest classifier was trained on the first 100 PCA components to distinguish OA from CA.

#### Baseline 3—2D Fast Fourier Transform-Convolutional Neural Network

**Figure 5:**
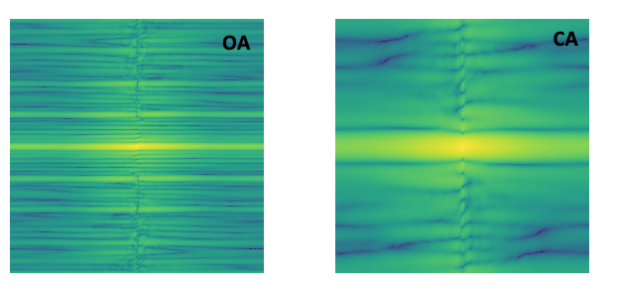
2D fast Fourier transform (2DFFT) of movement histograms for OA and CA. 2DFFT images of OA have a wider frequency range as compared with CA, as breathing effort during OA causes more fluctuation in the movement signal. 2DFTT: 2D fast Fourier transform; CA: central apnea; OA: obstructive apnea.

This method separates OAs from CAs based on the frequency-domain representation of movement histograms. The movement histograms of each event were concatenated together over time to form an image and then transformed to the frequency domain via 2DFFT with a constant size of 128×128. A sample of 2DFFT signal is shown in Figure 5 for a CA and an OA. A CNN (DarkNet19) [30] was trained on the obtained 2DFFT image to distinguish between OA and CA.

#### Validation

The data were divided into training and validation and test sets by the room in which the study was conducted to ensure the setup camera placement did not affect the algorithm performance. The recorded data of 21 participants (recorded in laboratory room number 1) were used in the training and validation sets. This set included 40 CAs and 313 OAs. The remaining 21 (recorded in laboratory room number 2) comprised the test set, which included 75 CAs and 299 OAs.

For the autocorrelation and movement histogram methods, classifier hyperparameters were tuned via 3-fold cross-validation on the training set. For the 3D-CNN (whole body) and 3D-CNN (chest and abdomen) methods, early stopping was based on performance on the validation set.

Performance of the head, chest, and abdomen bounding box detection was evaluated based on accuracy at the intersection over union values higher than 0.5 between predicted and manually annotated regions.

## Results

Data from 42 participants (27 men and 15 women) were collected for this study. Participants’ demographic information is shown in Table 2. None of the parameters were significantly different between the groups, except BMI with a *P* value of .04.

**Figure 6:**
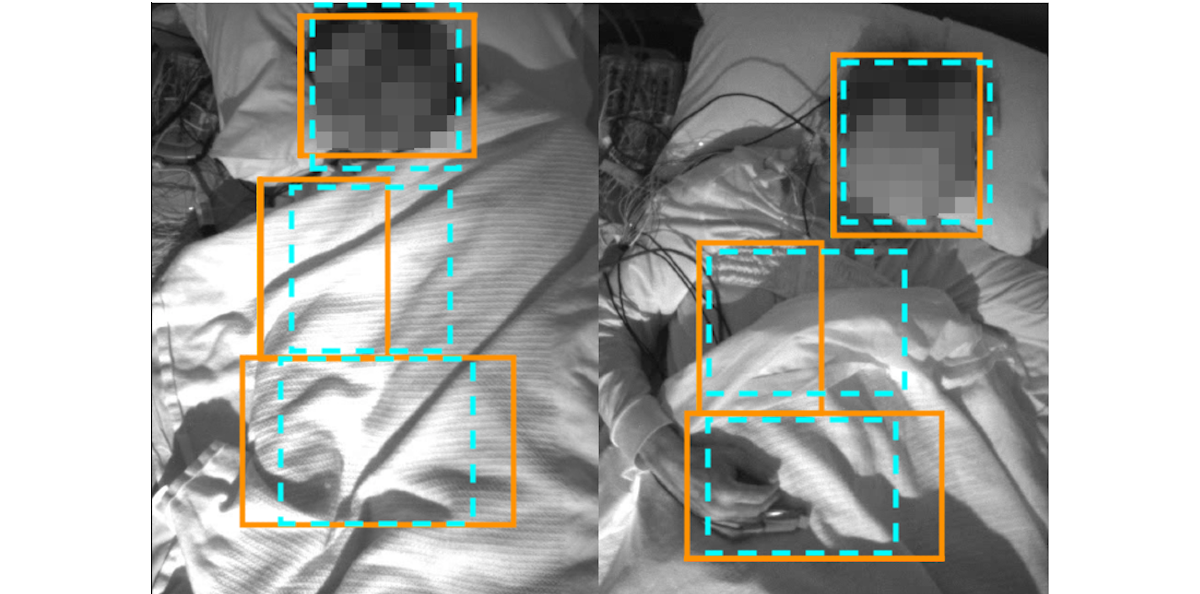
Sample chest, abdomen, and head detection results. Manually annotated and detected regions are shown in blue hashed line and orange solid line, respectively.

Figure 6 shows a sample image frame, as well as the manually marked and automatically detected bounding boxes, for the chest and the abdomen. The performance of the head, chest, and abdomen bounding box detection is quantified in Table 3. The head detection model obtained 92% accuracy, which was higher than the chest and abdomen detection models with accuracies of 83% and 67%, respectively.

Results of distinguishing OAs from CAs are shown in Table 4. Different classifiers obtained similar performance for the movement histograms method. For the sake of space, only results of the random forest classifier are shown in Table 4. The 3D-CNN model obtained the best performance with 95.4% accuracy and an *F*_1_ score of 88.7%.

**Table 2:** Participant demographics (N=42).

Characteristics^a^	Room 1 (test set), mean (SD)	Room 2 (train set), mean (SD)
Male	8 (13)	7 (14)
Age (years)	53 (15)	55 (13)
BMI^b^ (kg/m^2^)	28 (6)	32 (7)
Sleep efficiency (%)	73 (18)	75 (18)
REM^c^ sleep percentage (%)	16 (6)	15 (8)
Mean wake heart rate (bpm^d^)	66 (17)	71 (15)
Mean REM heart rate (bpm)	63 (18)	72 (12)
Minimum SaO_2_^e^	81 (9)	81 (7)
Mean SaO_2_	94 (3)	94 (3)
Number of OAs^f^ (events)	16 (35)	16 (23)
Number of CA^g^ (events)	4 (10)	2 (3)
AHI^h^ (events/hour)	24 (35)	29 (26)
Sleep duration (hour)	5 (1)	5 (1)

^a^Participants’ information calculated from the sleep reports of the overnight sleep study of participants annotated by sleep technicians.

^b^BMI: body mass index. BMI is different between the two rooms with a *P* value of .04.

^c^REM: rapid eye movement.

^d^bpm: beats per minute.

^e^SaO_2_: arterial oxygen saturation.

^f^OA: obstructive apnea.

^g^CA: central apnea.

^h^AHI: apnea-hypopnea index.

**Table 3:** Face, chest, and abdomen bounding box.

Detected object	Accuracy (%, at IoU^a^ >0.5), mean (SD)
Head	92 (11)
Chest	83 (14)
Abdomen	67 (15)

^a^IoU: intersection over union.

**Table 4:** Obstructive apneas versus central apneas: prediction performance of different models.

Method	Accuracy^a^ (%)	Precision^a^ (%)	Recall^a^ (%)	*F*_1_ score^a^ (%)
Autocorrelation	88.4	81.1	53.1	64.2
Histogram of movements	88.5	86.7	48.2	61.9
2DFFT-CNN^b^	89.7	69.1	75.6	72.3
3D-CNN^c^	95.4	88.2	89.3	88.7
3D-CNN chest and abdomen (annotated)	90.9	71.1	81.8	76.1
3D-CNN chest and abdomen (estimated)	89.3	72.1	76.0	74.0

^a^Accuracy, precision, recall, and *F*_1_ score indicate the ratio of correct prediction to the total number of data points, the ratio of correct positive prediction to the total positive prediction, the ratio of correct positive prediction to the total positive data, and the harmonic mean of precision and recall.

^b^2DFFT-CNN: 2D fast Fourier transform-convolutional neural network

^c^3D-CNN: 3D convolutional neural network.

## Discussion

### Principal Findings

**Figure 7:**
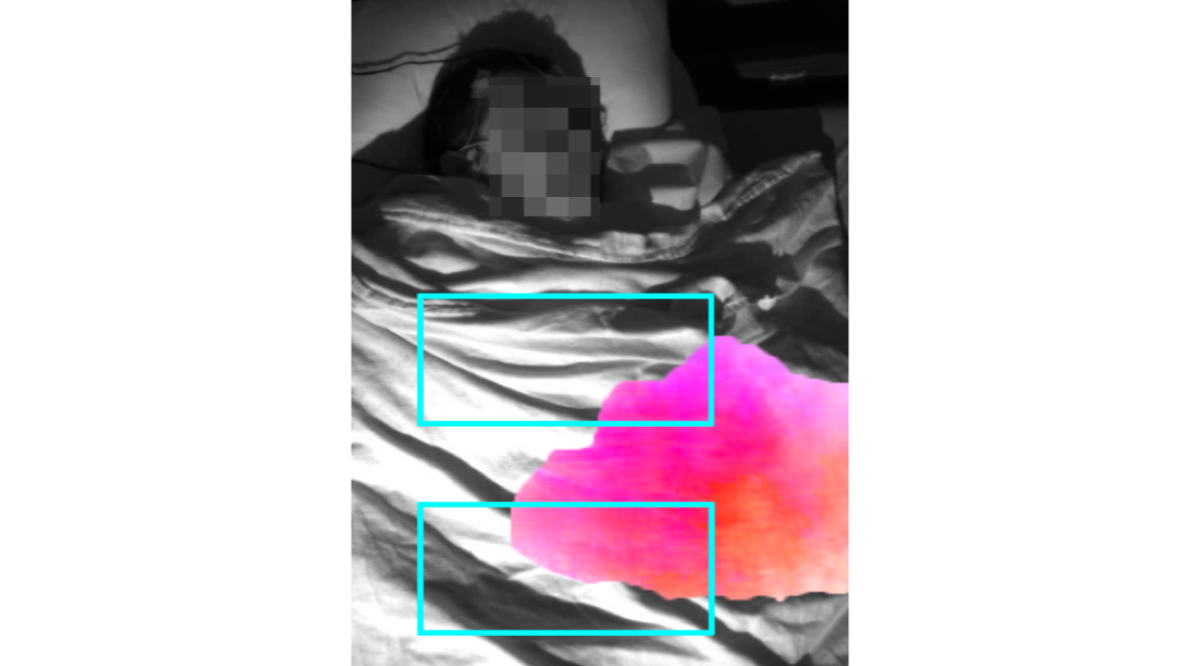
Annotated chest and abdomen regions do not capture a large area where most of the respiratory-related movement is visible. Manually annotated chest and abdomen regions are shown with blue boxes. Areas with large movement intensity (magnitude of the optical flow) are highlighted in pink.

The proposed 3D-CNN model outperformed all three baseline methods. We hypothesized that localizing the chest and abdomen in the video will increase the signal-to-noise ratio to improve performance. However, as shown in Table 4, applying 3D-CNN on the whole image frame obtained the best performance. Figure 7 illustrates how the use of a blanket may explain these results. The blanket propagates chest and abdominal movements outside of their respective detected (or annotated) regions. As a result, localizing the chest and abdomen locations removed part of the respiratory-related movement signal. In addition, accurate detection of abdomen location is challenging when the body is covered by a blanket; however, increasing the number of data points (>42) could potentially address this challenge. Attention mechanisms could also potentially be used to automatically identify image regions in which chest or abdomen movements are prominent.

### Limitations

The methods developed and evaluated in this study are the first to use computer vision to differentiate between OAs and CAs. Although the models were externally validated on data collected in a different room, a limitation of this study was that the same camera model and camera setup was used to collect data in both rooms. Remedying this limitation will involve external validation on a dataset that will be recorded under different conditions, for example, use of another camera model, different viewing angle, or a different camera distance from the bed. This will evaluate how the models trained here generalize to potential variations that might occur in real-life scenarios, for example, in the home. Another limitation of the current system is that it relies on the assumption that apneas were already segmented, for example, using previously developed vision-based methods [21]. Remedying this limitation will involve evaluation of how the combination of such methods with the models developed here will perform.

### Comparison With Prior Work

To address the challenges associated with PSG, there have been several investigations to develop convenient sleep apnea screening devices that can also distinguish CA from OA [14,15,28,31,32]. In a study proposed by Argod et al [15], pulse transit time technique was used to measure the delay between the R-wave on the electrocardiogram and a finger. They used the delay to visually classify CA from OA [15]. In another study, Park et al [31] designed an invasive implantable cardiac device to differentiate between CA and OA based on oscillation characteristics of the cardiac electrical activity. Luo et al [14] used the diaphragm electromyogram to track the activity of respiratory muscles to differentiate OA from CA. Thomas et al [28] used a single-lead electrocardiogram to classify OA from CA by measuring the elevated low-frequency coupling of heart rate variability and the fluctuations in the amplitude of the R-wave. These studies either are invasive or need the attachment of sensors to the body, which could be inconvenient and sensitive-to-motion artifacts and disrupt the user’s regular sleep pattern. A noncontact method to distinguish CA from OA will address these challenges.

In one attempt, Nandakumar et al [32] tracked body movements via smartphones. They used frequency-modulated continuous-wave transmissions to find the motion changes in body. Although their model counted the number of CAs and OAs, the performance of the model on localizing and distinguishing of apneas was not reported. Moreover, this study did not report cross-validation results.

### Conclusions and Future Works

This research project is the first vision-based noncontact method that differentiates sleep apneas by tracking body movements using IR videos. The developed algorithm was validated on 42 participants with various levels of sleep apnea severity. The algorithm performed well in distinguishing the OAs from the CAs. In this study, it was assumed that apneas were given. Future work can apply existing techniques [21] or novel CNN-based methods to identify apneas. Similar approaches can be used for noncontact assessment of respiration and respiratory effort during exercise testing in individuals who are using mechanical ventilators and infants with respiratory problems. Future work also involves collecting more data to perform an external validation of models developed here under varying conditions, as well as to improve chest and abdominal detection accuracy.

## Abbreviations

AHI: apnea-hypopnea index
CSA: central sleep apnea
CA: central apnea
CNN: convolutional neural network
CPAP: continuous positive airway pressure
2DFFT: 2-dimensional fast Fourier transform
IR: infrared
OSA: obstructive sleep apnea
OA: obstructive apnea
PCA: principal component analysis
PSG: polysomnography
RIP: respiratory inductance plethysmography
